# Asparagine Endopeptidase (δ Secretase), an Enzyme Implicated in Alzheimer’s Disease Pathology, Is an Inhibitor of Axon Regeneration in Peripheral Nerves

**DOI:** 10.1523/ENEURO.0155-20.2020

**Published:** 2021-01-05

**Authors:** Arthur W. English, Xia Liu, Olivia C. Mistretta, Patricia J. Ward, Keqiang Ye

**Affiliations:** 1 Department of Cell Biology, Emory University School of Medicine, Atlanta, GA 30322; 2 Department of Pathology and Laboratory Medicine, Emory University School of Medicine, Atlanta, GA 30322

**Keywords:** APP, asparagine endopeptidase, mouse, nerve, regeneration, tau

## Abstract

Asparagine endopeptidase (AEP) is a lysosomal protease implicated in the pathology of Alzheimer’s disease (AD). It is known to cleave the axonal microtubule associated protein, Tau, and amyloid precursor protein (APP), both of which might impede axon regeneration following peripheral nerve injury (PNI). Active AEP, AEP-cleaved fragments of Tau (Tau N368), and APP (APP N585) were found in injured peripheral nerves. In AEP null mice, elongation of regenerating axons after sciatic nerve transection and repair was increased relative to wild-type (WT) controls. Compound muscle action potentials (M responses) were restored in reinnervated muscles twice as fast after injury in AEP knock-out (KO) mice as WT controls. Neurite elongation in cultures of adult dorsal root ganglion (DRG) neurons derived from AEP KO mice was increased significantly relative to cultures from WT controls. In AEP KO mice exposed to 1 h of 20-Hz electrical stimulation (ES) at the time of nerve injury, no further enhancement of axon regeneration was observed. These findings support inhibition of AEP as a therapeutic target to enhance axon regeneration after PNI.

## Significance Statement

Axons in injured peripheral nerves can regenerate, but recovery from such injuries is poor. Asparagine endopeptidase (AEP) is an enzyme linked to the major pathologic features of Alzheimer’s disease (AD). We show here that it is highly expressed in injured peripheral nerves and both reduces the microtubule stabilizing effect of Tau on axons and degrades amyloid precursor protein (APP) in a manner that hinders any axon pathfinding role. Removing AEP by gene knock-out (KO) enhanced the elongation of regenerating axons in cut nerves and promoted an accelerated restoration of neuromuscular function. Inhibition of AEP is thus a target for development of novel strategies to treat peripheral nerve injuries (PNIs).

## Introduction

Although only ∼10% of >200,000 new traumatic peripheral nerve injuries (PNIs) that occur in the United States each year ever recover function (Frostick et al., 1998; Scholz et al., 2009), there is currently no widely used non-surgical treatment available. Enhancing axon regeneration has emerged as a goal for the development of new treatments following PNI (Höke and Brushart, 2010).

Following injury to a peripheral nerve, regenerative sprouts in the proximal segment lead to formation of regenerating axons that pass into a regeneration pathway. This process of elongation, which is heavily influenced by axon guidance molecules (Dun et al., 2019; Dun and Parkinson, 2020), involves distal addition of cytoskeletal elements, such as microtubules (Bamburg et al., 1986). Microtubules are polymers of α and β tubulin monomers and they can change dynamically between polymerization and de-polymerization states (Conde and Cáceres, 2009). The microtubule-associated protein, Tau, binds to microtubules and promotes their stabilization in growing axons (Drubin and Kirschner, 1986; Black et al., 1996). Asparagine endopeptidase (AEP; also known as δ-secretase or legumain) is a lysosomal cysteine protease that cleaves on the C terminal side of asparagine residues. It cleaves Tau at N255 and N368, thereby removing its microtubule-binding domain completely. AEP cleaves Tau *in vitro* and in human Alzheimer’s disease (AD) brains, abolishing its microtubule stabilizing function, inducing Tau aggregation, and triggering neurodegeneration (Zhang et al., 2014).

Another AEP substrate implicated in AD, amyloid precursor protein (APP), is often associated with axon pathfinding (Farah, 2012). It is found in growth cones (Sosa et al., 2013) and contains a receptor for the Slit family of glycoprotein axon guidance molecules on its extracellular domain (Wang et al., 2017). It also has been suggested to play a role as a co-receptor with several other axon guidance partners (Southam et al., 2019). Most of the ectodomain of APP can be cleaved by β-site APP cleaving enzyme 1 (BACE1 or β-secretase), removing its Slit binding site. After peripheral nerve crush, increasing BACE1 activity inhibits regeneration (Tallon et al., 2017), while in mice null for BACE1, regeneration is enhanced (Farah et al., 2011). Subsequent to BACE1 cleavage of APP, the C-terminal fragment of APP can be cleaved by Ɣ-secretase to form amyloid-β protein (Aβ). When applied to cultures of cortical neurons, Aβ stimulated growth cone collapse and neurite retraction (Kuboyama, 2018). In the brain, AEP cleaves APP at N585, removing its Slit binding site but also shortening the long extracellular domain of APP, thereby reducing steric hindrance to BACE1 activity and enhancing Aβ generation (Zhang et al., 2015). Thus, increased AEP activity after PNI could inhibit axon regeneration both by interfering with the guidance role of APP and via increased production of Aβ.

It is not known whether enzymatically active AEP is found in injured nerves, but if it is, it could impair axon regeneration by its actions on both Tau and APP. If removed or inhibited, AEP could result in enhanced axon regeneration. One goal of this study was to investigate the activity of AEP in a widely used model of PNI and to evaluate axon regeneration in this model in mice in which AEP is knocked out.

Brief electrical stimulation (ES) has been used to enhance axon regeneration after PNI. Its success depends on an increase in signaling by brain-derived neurotrophic factor (BDNF) and its TrkB receptor in the regenerating axons (Gordon and English, 2016). Because BDNF-TrkB signaling can drive an Akt-mediated block of AEP activity, both *in vitro* and in mouse brains (Wang et al., 2018), we hypothesized that the effectiveness of ES in promoting axon regeneration after PNI is the result of AEP inhibition. Thus, a second goal of this study was to evaluate whether ES would enhance axon regeneration in mice null for AEP.

## Materials and Methods

### Experimental design and statistical analysis

When comparing parametric data from more than two groups, a one-way ANOVA was used. If the omnibus result of that analysis was significant (*p* < 0.05), groups were compared in a *post hoc* manner using Tukey’s HSD test. For non-parametric analyses (distributions of axon profile lengths), comparisons were made in a pairwise manner using a Kolmogorov–Smirnov (K–S) two-sample test. For time-series data, comparisons were made using multiple linear regression analysis. Significance of all statistical comparisons was set at *p* < 0.05. All statistical comparisons were performed using GraphPad Prism.

### Animals

All mice used in both *in vivo* experiments and as a source of cells for *in vitro* studies were on a mixed C57BL6-129/Ola background. Mice null for AEP [AEP^−/−^ or AEP knock-out (KO)] are the same as those described elsewhere (Shirahama-Noda et al., 2003). Animals used in experiments were from the F_1_ generation of crosses between heterozygous (AEP^+/−^) animals. All mice used were genotyped by Transnetyx Inc. from tail DNA before inclusion in experiments. All experimental methods were approved by the Institutional Animal Care and use Committee of our institution (Protocol 201800101). All experiments conformed to the Policies on the Use of Animals and Humans in Neuroscience Research of the Society for Neuroscience.

For analyses of the lengths of regenerating axons, AEP^−/−^ mice were bred with mice in which a subset of motor and sensory axons in peripheral nerves is marked completely by the expression of yellow fluorescent protein (YPF). In the these SLICK-A mice, YFP and a tamoxifen-inducible cre recombinase are expressed under the control of the thy-1 promoter (Young et al., 2008). No cre-dependent transgenes were expressed in these mice and no mice used were ever exposed to tamoxifen. They were used only to mark regenerating axons that expressed YFP.

### Surgical methods

Under general anesthesia (ketamine 80 mg/kg/xylazine 10 mg/kg) the sciatic nerve was exposed in the posterior thigh. Two types of transection and repair of the sciatic nerve were performed. In most experiments, a simple end-to-end anastomosis was performed. A small (∼1 × 2 mm) piece of SILASTIC film (Dow Corning #501-1) was placed beneath the exposed sciatic nerve, just proximal to the branch point of the sural nerve. The intact nerve was then secured to the film with 1 μl of fibrin glue: a mixture of fibrinogen and thrombin (1:2, Sigma-Aldrich; de Vries et al., 2002; MacGillivray, 2003). Once the glue had set, the nerve was cut in the center of the film using sharp scissors. The cut ends of the nerve remained relatively well-aligned and were further secured with another microliter of fibrin glue. Surgical wounds were then closed in layers and the animals allowed to recover fully from the anesthesia before returning them to their cages. Numbers of animals exposed to this nerve repair procedure are given in the different groups below.

In 24 additional mice, measurement of regenerating axon profile lengths was used as an outcome measure and a slightly different nerve transection and repair method was employed, using in mice on the SLICK-A background described above. In anesthetized animals, the sciatic nerve was exposed, attached to a patch of SILASTIC film, and cut, as described above. The distal segment of the nerve was removed from the patch and replaced by a graft; a 15-mm-long segment of sciatic nerve harvested from an anesthetized littermate that did not express YFP. The exposed segments of nerves were aligned on the film and secured in place using fibrin glue. The graft was not connected to the distal segment of the cut sciatic nerve, but was draped along the length of the tendocalcaneus, beneath the hamstring fascia. Surgical wounds were then closed in layers and the animals allowed to recover fully from the anesthesia before returning them to their cages.

### Axon profile length analysis

Mice used as hosts in these experiments were all positive for the SLICK-A transgene and one of three AEP genotypes (AEP^+/+^, AEP^+/−^, or AEP^−/−^). All animals used were on the same background strain. Eight AEP KO (SLICK-A/AEP^−/−^) mice (four males and four females), six mice heterozygous for knock-out of the AEP gene (SLICK-A/AEP^+/−^), and six wild-type (WT; SLICK-A/AEP^+/+^, three males and three females in each group) were studied. Mice serving as nerve graft donors in these experiments all were matched to the genotype of the host mouse but were littermates that did not express the SLICK-A transgene, and thus did not express YFP. Grafts harvested from these mice thus served as a strain-matched dark background against which YFP+ regenerating axons could be visualized. To evaluate whether any effect of AEP activity on axon elongation was from cells in the pathway surrounding regenerating axons, cut nerves from four additional SLICK-A/AEP^+/+^ mice were repaired with grafts from AEP^−/−^ donors.

Two weeks after nerve transection and repair with grafts, animals were euthanized with Euthasol injection and the repaired nerves and attached grafts were removed and fixed overnight by immersion in PLP solution. Excess connective tissue and residual fibrin glue was carefully removed by dissection and the nerves were mounted onto microscope slides and coverslipped with Vectashield (Vector Laboratories). Edges of the cover slips were secured using clear nail polish.

Whole mounted nerves were imaged using a confocal microscope (Leica S8). Stacks of optical sections 10 μm thick were obtained through the entire thickness of the nerve and stacks through contiguous regions of the nerve were stitched together, in register, using the Leica software. The result was a three-dimensional reconstruction of the nerve repair site and the entire attached graft. Lengths of profiles of regenerating axons in the graft were measured in these stacks from their distal tips to the surgical repair site. The distributions of axon profile lengths in the different groups of mice were compared in a pairwise manner using a K–S two-sample test. In addition, average median axon profile lengths and a sprouting index (the ratio of the number of YFP+ regenerating axons in the grafts to the number of YFP+ axons 1 mm proximal to the injury site) were compared between the groups using ANOVA. Significance of differences was determined using paired *post hoc* testing (Tukey).

### Electrophysiology

The extent of functional muscle reinnervation was evaluated before and at two-week intervals for up to 14 weeks after sciatic nerve transection and end-to-end repair. Five male and five female AEP^−/−^ mice were studied and compared with five AEP^+/+^ mice (three males, two females). In each experiment, mice were anesthetized with isoflurane and maintained at a surgical plane throughout the procedure. A small incision was made in the proximal thigh to expose the sciatic nerve as it exits the pelvis. Two monopolar needle electrodes (Ambu Inc.), spaced 1 mm apart, were placed beneath the nerve and used to deliver short (0.3 ms) monophasic constant voltage stimulus pulses to the nerve. Muscle activity evoked by this stimulation was recorded from bipolar fine wire electrodes (Basmajian and Stecko, 1963) placed into the lateral gastrocnemius (GAST) and tibialis anterior (TA) muscles using a 25-G 5/8” hypodermic needle. The electrodes were constructed from enamel-coated stainless-steel wires (California Fine Wire Company, MO#M468240), in which the insulation was removed from the distal 1 mm of the recording tips.

Data collection was performed using custom designed Labview software. Ongoing full wave rectified EMG activity was monitored continuously and when the average activity over a 20-ms interval was within a user-defined voltage window, a stimulus was delivered to the sciatic nerve via the needle electrodes. The EMG activity, sampled at 10 kHz, was captured from 20 ms before the stimulus until 50 ms after the stimulus and recorded to disk. Stimulus intensity was then increased until a maximal EMG response was noted from each muscle. To avoid fatigue, stimuli were delivered no more frequently than once every 3 s.

Stimulation of the sciatic nerve resulted in the recording of direct muscle responses, or M responses, produced by the activation of motor axons innervating either GAST or TA. The magnitude of the largest evoked M response was assumed to be a measure of the extent of motor innervation of the muscle. For each triphasic M response recorded, the average rectified voltage within a user defined window was measured. In reinnervated muscles, the M response latency is initially longer than that found in intact animals (Chen et al., 2010; Boeltz et al., 2013), so that these windows had to be adjusted in analysis of results of each experiment to include the entire M wave. The maximum M response (M_MAX_) amplitude measured at different times after sciatic nerve transection and repair was then scaled to the M_MAX_ amplitude recorded before injury, as a measure of the extent of recovery. Differences in the recovery of these scaled M_MAX_ values in the different groups of mice were evaluated in a pairwise manner using multiple linear regression analysis (Boeltz et al., 2013). Significance of differences in the slopes of the regression lines between pairs of groups was evaluated using ANOVA.

### Cell culture experiments

Primary cultures of adult dorsal root ganglion (DRG) cells were prepared. In older than two-month-old mice of both sexes euthanized with an overdose of isoflurane, DRGs were extracted and placed in cold HBSS (Corning), incubated in dispase (2.5 μ/ml Sigma-Aldrich) and collagenase (200 μ/ml Worthington Biochemical) in a 37°C bead bath for 45 min with gentle agitation applied every 15 min, and treated in 37°C DNase (Worthington Biochemical) for 2.5 min before addition of room temperature HBSS. Cells were triturated, the HBSS was removed, and the cells were resuspended in neurobasal medium A (NB-A; Invitrogen) containing 2% B-27 (Invitrogen), 1% penicillin/streptomycin (Lonza Biowhittaker), and 1% Glutamax (Invitrogen). Cells were seeded at a density of 1000 cells/well in dishes coated with laminin (0.2 mg/ml, Thermo Fisher Scientific) and poly-l-lysine (2 mg/ml, Sigma-Aldrich).

Seventy-two hours after plating, cells were fixed with periodate-lysate-paraformaldehyde (PLP) solution (McLean and Nakane, 1974) and incubated for 14–16 h with anti-tubulin β−3 antibody (Biolegend, 1:1000), followed by goat anti-mouse antibody conjugated to Alexa Fluor 555 (Invitrogen). Cells were imaged at 20× magnification and the longest neuritic processes from each cell were measured using the Fiji software package (ImageJ). At least 60 cells per treatment were measured from cultures from each animal. Mean lengths were compared between the three AEP genotypes (AEP^+/+^, AEP^+/−^, and AEP^−/−^) using ANOVA and *post hoc* (Tukey) paired testing.

### Immunoblotting

To evaluate the expression and enzymatic effects of AEP, extracts from sciatic nerves of intact AEP^+/+^ or AEP^−/−^ mice and mice at different times after sciatic nerve transection and repair were analyzed using immunoblots. Animals were euthanized with Euthasol solution and a ∼2-mm-long segment of the sciatic nerve containing the surgical repair site was harvested and frozen immediately in liquid nitrogen. For intact animals, a similar segment of the nerve at a similar location was harvested.

The frozen nerves were lysed in Laemmli buffer (62.5 mm Tris-HCl, pH 6.8, 10% glycerol, 2% SDS, 5% 2-mercaptoethanol, and 0.005% bromophenol blue) and followed by homogenization with sonication. After heating at 98°C for 5 min, similar amounts of tissue lysate from different nerves were separated by SDS-PAGE, transferred to nitrocellulose membranes, and probed with the following antibodies to detect the corresponding targets: anti-AEP (catalog #93627s, Cell signaling), anti-APP (catalog #MAB348MI, Fisher), anti-Tau (catalog #MAB361, Millipore), anti-β-Actin (catalog #A1978, Sigma), anti-APP N585 (Covance custom made; Wang et al., 2018; 1:1000 NF), anti-Tau N368 (Covance custom made; Zhang et al., 2014; 1:1000 NF). Antibody binding was detected using appropriate peroxidase-conjugated secondary antibodies and visualized using enhanced chemiluminescence. Quantitative analyses of immunoreactivity (IR) in blots were performed using FIJI (ImageJ) software. Intensity of expression of AEP, Tau N368, Tau 5, and APP N585, adjusted for loading, were compared as fold changes relative to samples from intact nerves of WT mice or cut and repaired nerves from AEP KO mice. Statistical comparisons were made using ANOVA and *post hoc* (Tukey) paired testing, where appropriate.

### ES

Application of 1 h of ES at the time of nerve repair has been shown to enhance subsequent axon regeneration in a BDNF-dependent and TrkB-dependent manner (Al-Majed et al., 2004; Geremia et al., 2007; Gordon and English, 2016). Based on reports in the literature, BDNF-TrkB signaling can result in inhibition of the activity of AEP in the brain. To test the hypothesis that ES would not further enhance the regeneration of axons in AEP KO mice, sciatic nerve transection and repair by end-to-end anastomosis was performed bilaterally, as described above, but just before nerve transection, the sciatic nerve on one side of each mouse was electrically stimulated, as above, for 1 h at 20 Hz (Al-Majed et al., 2000). Stimulus intensity was set to be twice that needed to evoke an observable M response in the innervated GAST muscle. In one group of mice (*N* = 8; four WT and four AEP KO), nerves were harvested one week after nerve repair surgery and processed for immunoblotting, as described above, to evaluate the effectiveness of ES in promoting BDNF-TrkB signaling in WT and AEP KO mice and in moderating the impact of AEP on Tau and APP.

In a second group of mice, M responses were evoked in the reinnervated muscles four weeks after nerve injury, as described above, and the amplitudes of the largest response (M_MAX_) were measured. The amplitudes of these responses were expressed as a function of similar measures recorded before nerve transection. Eight AEP KO mice (four males, four females) and eight strain-matched WT control mice (four males, four females) were treated with 1 h of ES. For comparison, similar data were collected from eight (four male, four female) unstimulated (US) AEP KO and eight US WT (four male, four female) mice. Significance of differences in M_MAX_ amplitudes between groups was performed using ANOVA and *post hoc* (Tukey) paired testing.

## Results

### AEP cleaves Tau and APP in cut and repaired peripheral nerves

**Figure 1.**
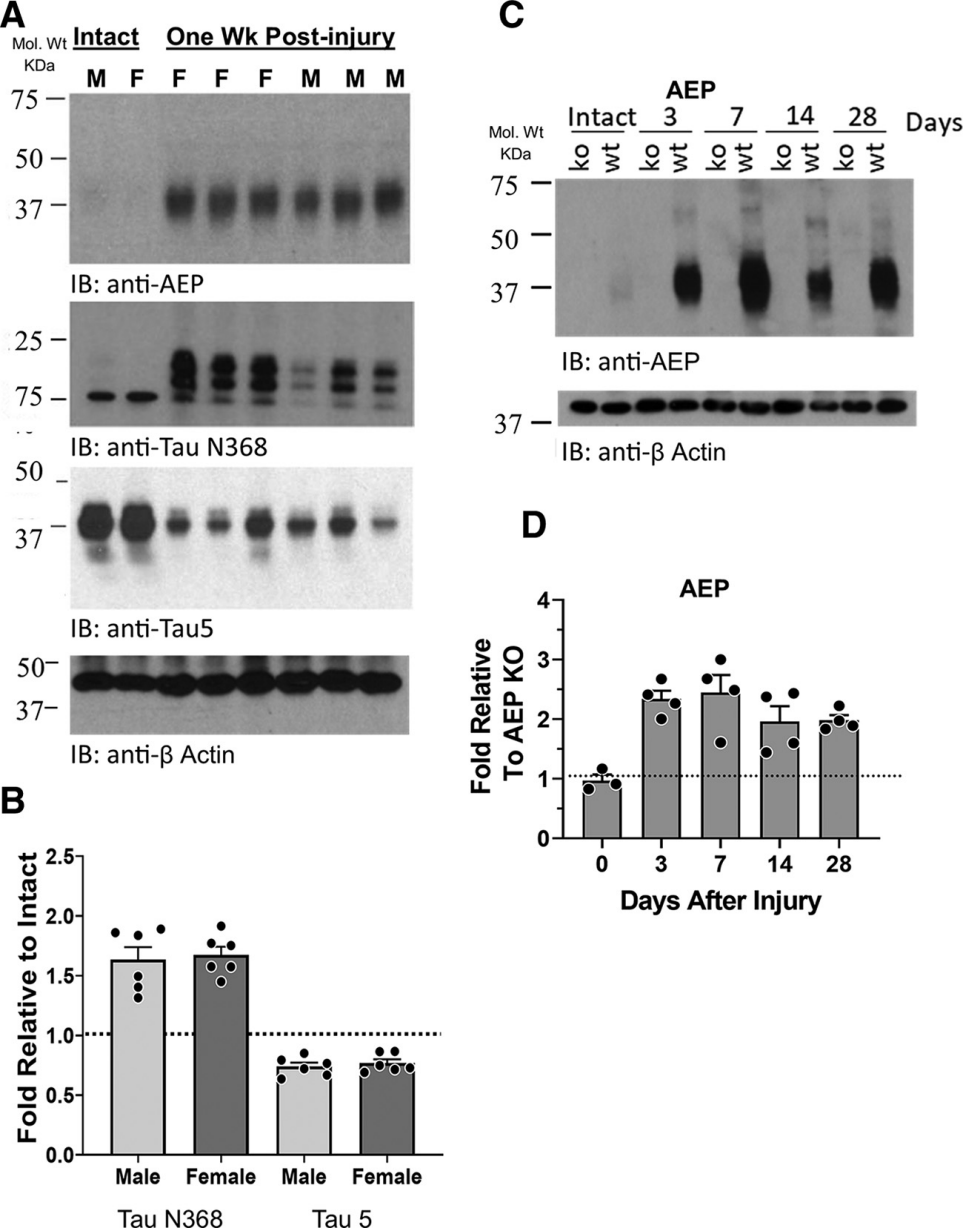
***A***, Immunoblots of extracts of sciatic nerves from intact mice and from nerves cut and repaired one week earlier. IR to AEP (top) is noted by the band at 37 kDa, representing enzymatically active AEP. IR to the AEP-cleaved fragment of Tau (Tau N368) and to full-length Tau (Tau 5) are shown below. ***B***, Quantitative analysis of differences in expression of Tau N368 and Tau 5 between intact nerves and cut and repaired nerves, relative to β actin controls, are shown for a group of male and female mice as mean fold (±SEM) intensities. The horizontal dashed line at unity marks the amount of IR found in intact mice. ***C***, Changes in AEP IR are shown at different times after sciatic nerve transection and repair in WT and AEP KO mice. ***D***, Quantitative analysis of AEP expression in sciatic nerves at different times after nerve transection and repair. Mean (±SEM) fold expression of AEP IR, relative to that found in AEP KO mice at the same postinjury times are shown. The horizontal dashed line at unity marks the amount of IR found in AEP KO mice.

The presence of AEP in cut and repaired peripheral nerves and the extent of its enzymatic activity on two known AEP substrates, Tau and APP, were studied using immunoblotting. IR to AEP was increased dramatically in sciatic nerves one week after nerve transection and repair (Fig. 1*A*, AEP). To become enzymatically active, AEP must be cleaved at both its C-terminal and N-terminal ends, resulting in a decrease in molecular weight from ∼60 to ∼37 kDa (Li et al., 2003). Relative to loading controls (β actin), expression of the enzymatically active (∼37 kDa) form of AEP is significantly increased one week after sciatic nerve transection and repair (Fig. 1*A*, AEP). This active AEP cleaved Tau in the cut and repaired sciatic nerves, as noted by increased IR to an antibody that recognizes an epitope revealed by AEP cleavage of Tau protein at N368 (Zhang et al., 2014; Fig. 1*A*, Tau N368), and a significant decrease in full-length Tau, as detected using antibody Tau 5 (Fig. 1*A*, Tau 5). Expression of Tau N368-IR was significantly increased (ANOVA, *F*_(3,12)_ = 21.86, *p* = 0.38) in injured nerves of both male and female mice, relative to samples of nerves from intact animals. The fold increase in Tau N368-IR in injured nerves, relative to intact nerves, was not significantly different in males than females (unpaired *t* test, *t* = 0.3614, df = 10, *p* = 0.38; Fig. 1*B*). The decrease in Tau 5-IR in injured nerves was significant in both sexes (ANOVA, *F*_(2,11)_ = 9.150, *p* < 0.01, *post hoc p* < 0.01 for both; Fig. 1*B*). No significant sex difference was found.

The time course of this postinjury increase in AEP is shown in immunoblots in Figure 1*C* and quantified in Figure 1*D*. In intact animals, no significant difference in AEP-IR was found when WT mice and mice null for AEP (AEP KO) mice were compared (fold relative to AEP KO = 1.0), suggesting again that significant AEP expression is absent from intact nerves. As early as 3 d after sciatic nerve transection and repair, a greater than 2-fold increase in AEP-IR was found, and the extent of this significant increase (ANOVA *F*_(4,14)_ = 6.919, *p* < 0.01, *post hoc p* < 0.03) persisted for at least 28 d.

**Figure 2.**
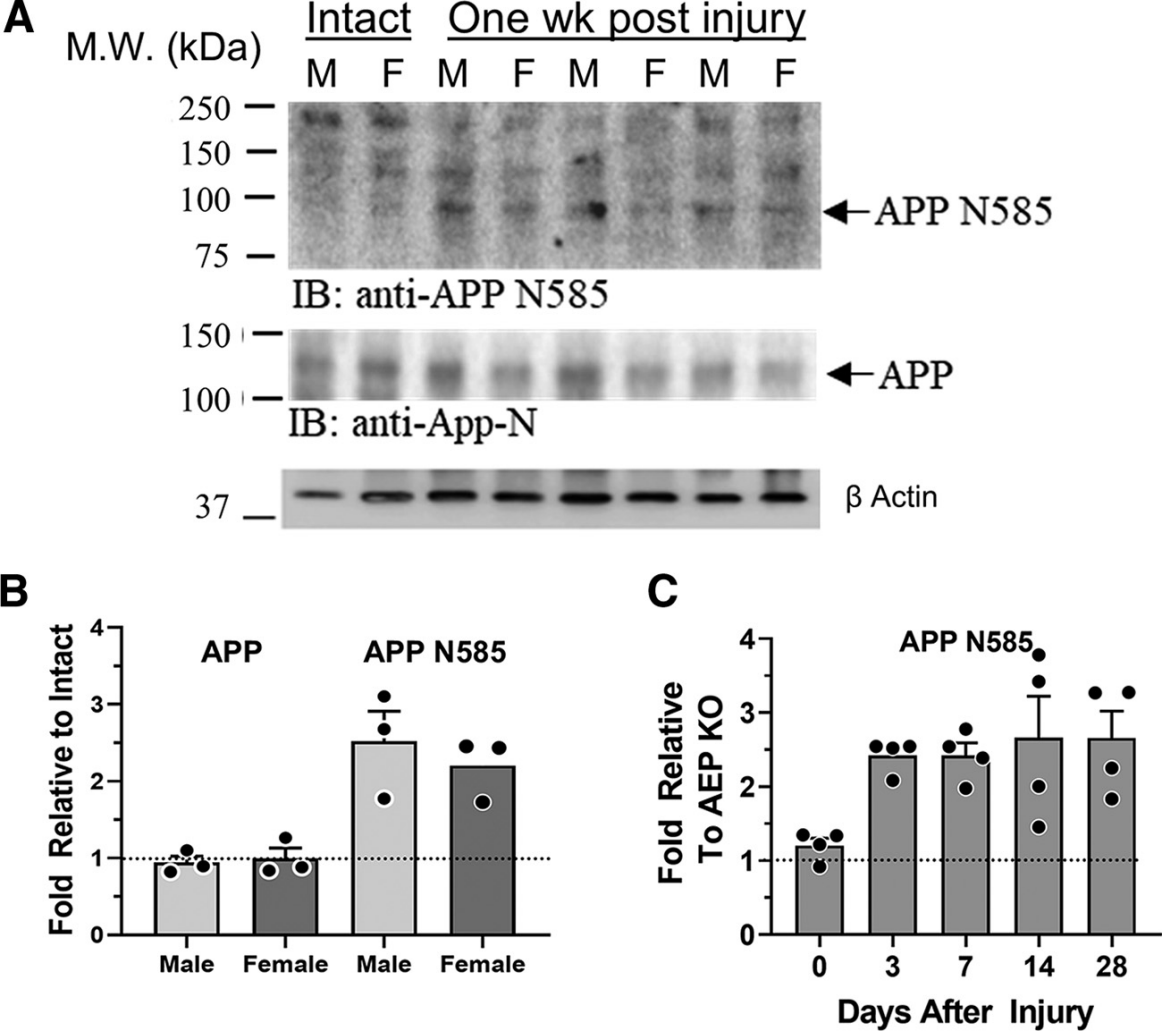
***A***, Immunoblots of extracts of sciatic nerves from intact mice and from nerves cut and repaired one week earlier. IR to an antibody recognizing the product of APP produced by AEP cleavage, APP N585 (top), and full-length APP (bottom) are shown. ***B***, Quantitative analysis of differences in expression of full-length APP (left) and APP N585 (right) between intact nerves and cut and repaired nerves, relative to β actin controls, are shown for a group of male and female mice as mean fold (±SEM) intensities. The horizontal dashed line at unity marks the amount of IR found in Intact mice. ***C***, Time course of expression of APP N585 in cut and repaired sciatic nerves. The horizontal dashed line at unity marks the amount of IR found in AEP KO mice.

The increase in active AEP in cut and repaired nerves was also accompanied by a cleavage of APP, as shown by IR to an epitope (N585) revealed by such cleavage (Wang et al., 2018) in injured nerves (Fig. 2*A*,*B*, APP N585). There was no significant change in expression of the full-length APP in the injured nerves (Fig. 2*B*, APP). As noted above, no significant APP N585 was found in intact nerves of WT mice or in intact or injured nerves from AEP KO mice, but it appeared rapidly and persisted at a significantly high level (ANOVA, *F*_(4,15)_ = 3.759, *p* < 0.03, *post hoc p* < 0.02) for at least 28 d after sciatic nerve transection and repair (Fig. 2*C*).

### Neurite elongation is increased in neurons cultured from AEP KO mice

**Figure 3.**
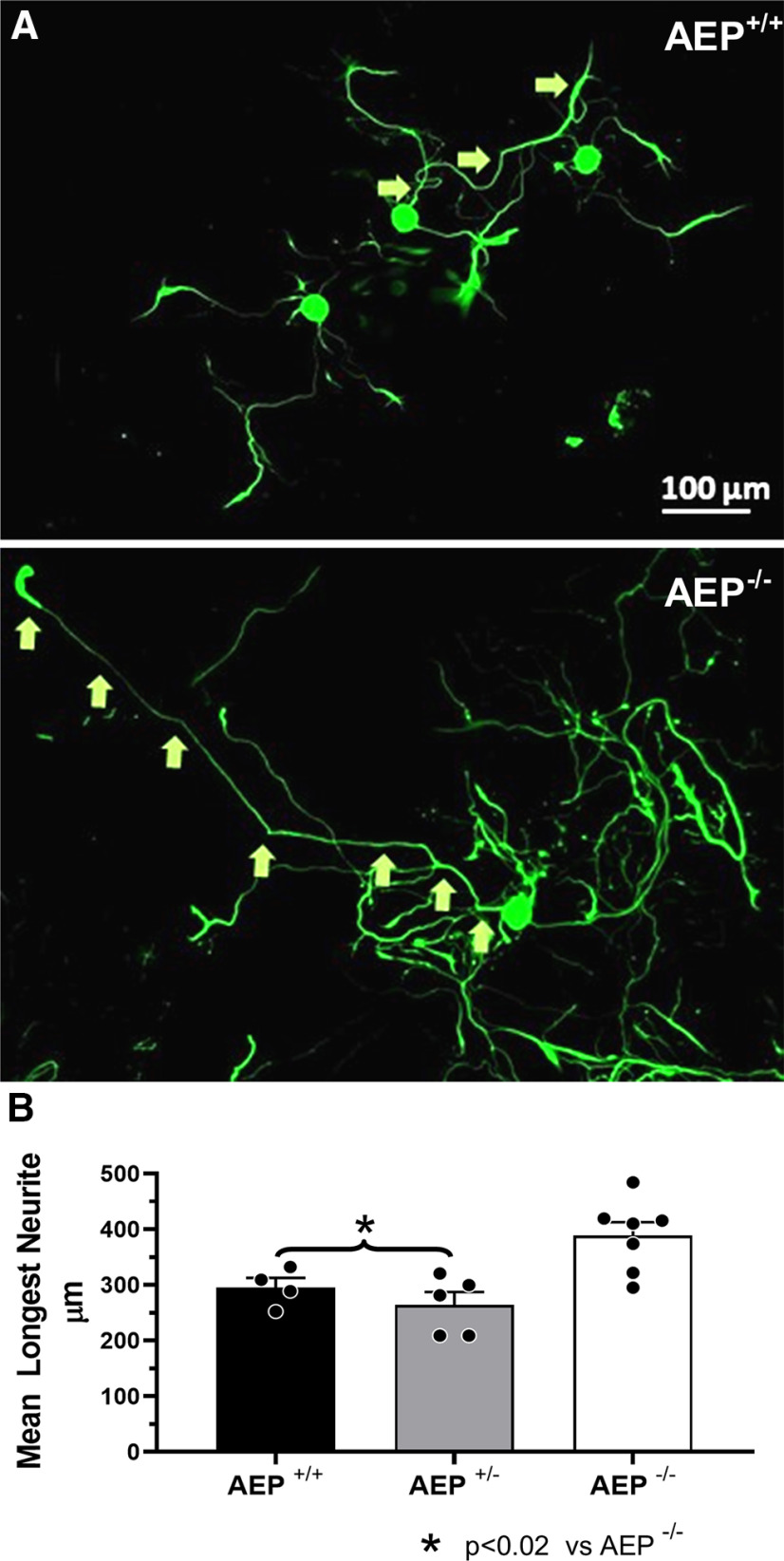
Neurite length was increased in cultured neurons from AEP^−/−^ mice. ***A***, Examples of neurons cultured from AEP^+/+^ (top) and AEP^−/−^ (bottom) mice. The identity of longest neurites of individual neurons is indicated by arrows. Scale bar: 100 μm. ***B***, Mean (±SEM) length of the longest neurites in cultures of adult DRG neurons from AEP^+/+^, AEP^+/−^, and AEP^−/−^ mice measured at 72 h *in vitro*.

We studied the elongation of neurites in primary cultures of adult DRG neurons derived from AEP^+/+^, AEP^+/−^, and AEP^−/−^ mice. In each culture we measured the longest neurites of at least 60 neurons 72 h after plating. Examples of neurons measured in cultures from AEP^+/+^, and AEP^−/−^ mice are shown in Figure 3*A*. Mean (±SEM) lengths of neurites in cultures from the different genotypes are shown in Figure 3*B*. Using a one-way ANOVA, a significant difference was found (*F*_(2,13)_ = 8.413, *p* < 0.01) between groups. Using *post hoc* paired testing significant differences were found between cultures from AEP^−/−^ mice and all others (*p* < 0.018 vs AEP^+/+^, *p* < 0.002 vs AEP^+/−^). No significant difference in neurite length was found between cultures from AEP^+/+^ and AEP^+/−^ mice.

### Axon regeneration is enhanced in AEP^−/−^ mice

**Figure 4.**
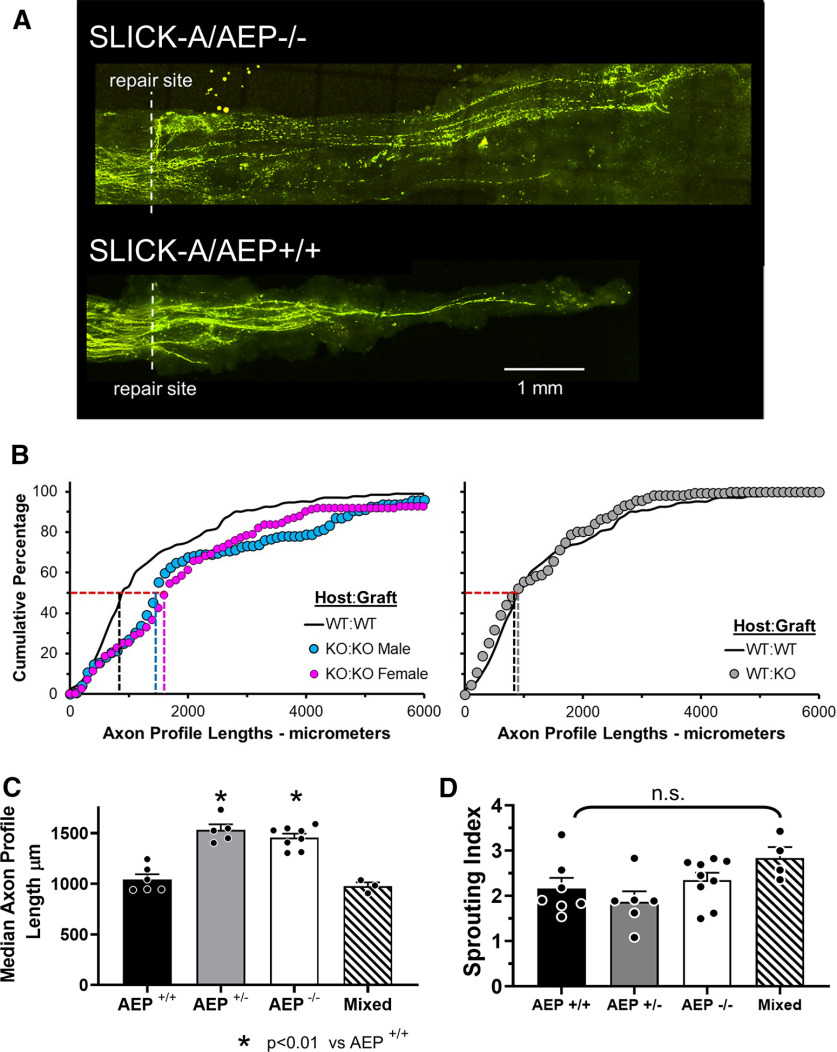
Elongation of regenerating axons is enhanced in AEP KO mice. ***A***, Examples of confocal images of sciatic nerves in SLICK-A mice that had been cut and repaired two weeks earlier with a segment of nerve harvested from a littermate that did not express YFP. Note more longer axons are found in the SLICK-A/AEP^−/−^ mouse (top). ***B***, Distributions of YFP+ regenerating axon profile lengths in male and female SLICK-A/AEP^−/−^ mice (KO:KO) and SLICK-A/AEP^+/+^ (WT:WT) mice measured two weeks after sciatic nerve transection and repair are shown as cumulative frequency histograms (left). Data from nerves from WT (SLICK-A/AEP^+/+^) mice repaired with grafts from AEP^−/−^ mice (WT:KO) in comparison to those from WT:WT nerves are shown in the graph on the right. Each set of symbols represents mean values in each bin (*N* = 5 except for WT:KO, where *N* = 4). Vertical dashed lines mark the lengths at the 50th percentile, the median for each distribution. ***C***, Average (±SEM) median lengths of regenerating axon profiles two weeks after sciatic nerve transection and repair in mice in which neither, both, or one copy of the gene for AEP had been knocked out, and when WT nerves were repaired with grafts from KO mice (mixed). ***D***, Mean (±SEM) sprouting index in mice of the three genotypes studied and mixed repair nerves. No significant differences were observed.

We crossed AEP KO mice with SLICK-A mice to study regenerating axon elongation. In the SLICK-A mice, a subset of sensory and motor axons in peripheral nerves is marked completely by YFP (Young et al., 2008). By repairing nerves with grafts from AEP-genotypically matched, but YFP negative littermates, the YFP+ regenerating axons could be visualized against a dark background using confocal microscopy. Images of nerves from SLICK-A/AEP^−/−^ and SLICK-A/AEP^+/+^ host mice two weeks after sciatic nerve transection and repair are shown in Figure 4*A*.

We measured the lengths of profiles of YFP+ regenerating axon profiles in these nerves, from their tips to the surgical repair site, in stacks of confocal images. The distributions of these lengths in male and female SLICK-A/AEP^−/−^ (KO) host mice and SLICK-A/AEP^+/+^ (WT) host mice are shown as cumulative histograms in Figure 4*B*, left panel. Because there is no sex difference in YFP+ regenerating axon profile lengths in untreated SLICK-A mice (Wilhelm et al., 2012), we combined data from males and females in the control group (WT:WT) for this graph. The distribution of lengths from SLICK-A/AEP^−/−^ host mice of both sexes are shifted significantly (K–S test, D = 0.4574, *p* < 0.01 for females; D = 0.3511, *p* < 0.01 for males) to the right of from SLICK-A/AEP^+/+^ host control mice, indicating that longer axon profile lengths were encountered in AEP KO mice of both sexes. No significant difference was found in these distributions when comparing male vs female AEP^−/−^ mice (K–S test, D = 0.1702, not significant).

We compared average median axon profile lengths in all these groups and in SLICK-A/AEP^+/−^ mice. Average median axon profile lengths (±SEM) for the different groups studied are shown in Figure 4*C*. We first performed a two factor ANOVA (sex vs genotype) and found a significant effect of genotype (*F*_(2,9)_ = 15.25, *p* < 0.01), but no significant effect of sex (*F*_(2,9)_ = 1.67, not significant) or of interaction (*F*_(4,9)_ = 1.58, not significant) between sex and genotype. Thus, we combined data from males and females for subsequent analysis. The results of one-way ANOVA of these data were significant (*F*_(2,15)_ = 12.25, *p* < 0.01). Using *post hoc* paired testing, significant (*p* < 0.01) differences were found between WT (SLICK-A/AEP^+/+^) mice and mice with either one or both AEP alleles deleted (Fig. 4*C*). No significant difference was found between homozygous and heterozygous AEP KO mice. Reducing or eliminating AEP expression resulted in enhanced axon regeneration.

We also analyzed axon profile lengths two weeks after cut nerves in SLICK-A:AEP^+/+^ mice were repaired with a segment of the same nerve from an AEP^−/−^ mouse. The distribution of axon profile lengths in these nerves is nearly identical to that of the control group (WT:WT), as shown in Figure 4*B*, right panel. Average (±SEM) median axon profile lengths are shown in Figure 4*C*. In these mixed repair nerves, neither measure of axon elongation was significantly different from those found in controls (WT:WT). Eliminating AEP in the pathway surrounding regenerating axons had no effect on elongation of regenerating axons.

The longer regenerating axons in SLICK-A:AEP^+/−^ and SLICK-A:AEP^−/−^ animals could be the result of fewer regenerating axons or regenerating axons that branch less. There was no significant difference in the numbers of YFP+ regenerating axons between the three genotypes (data not shown) so we evaluated the branching of regenerating axons. A sprouting index was calculated for each mouse studied. It is the ratio of the number of YFP+ axons found in the grafts to the number of YFP+ axons 1 mm proximal to the surgical repair site. Mean (±SEM) values of sprouting indices are shown for the different groups in Figure 4*D*. Using a one-way ANOVA, no significant differences were found.

### Compound muscle action potentials (M responses) are restored faster in AEP^−/−^ mice

**Figure 5.**
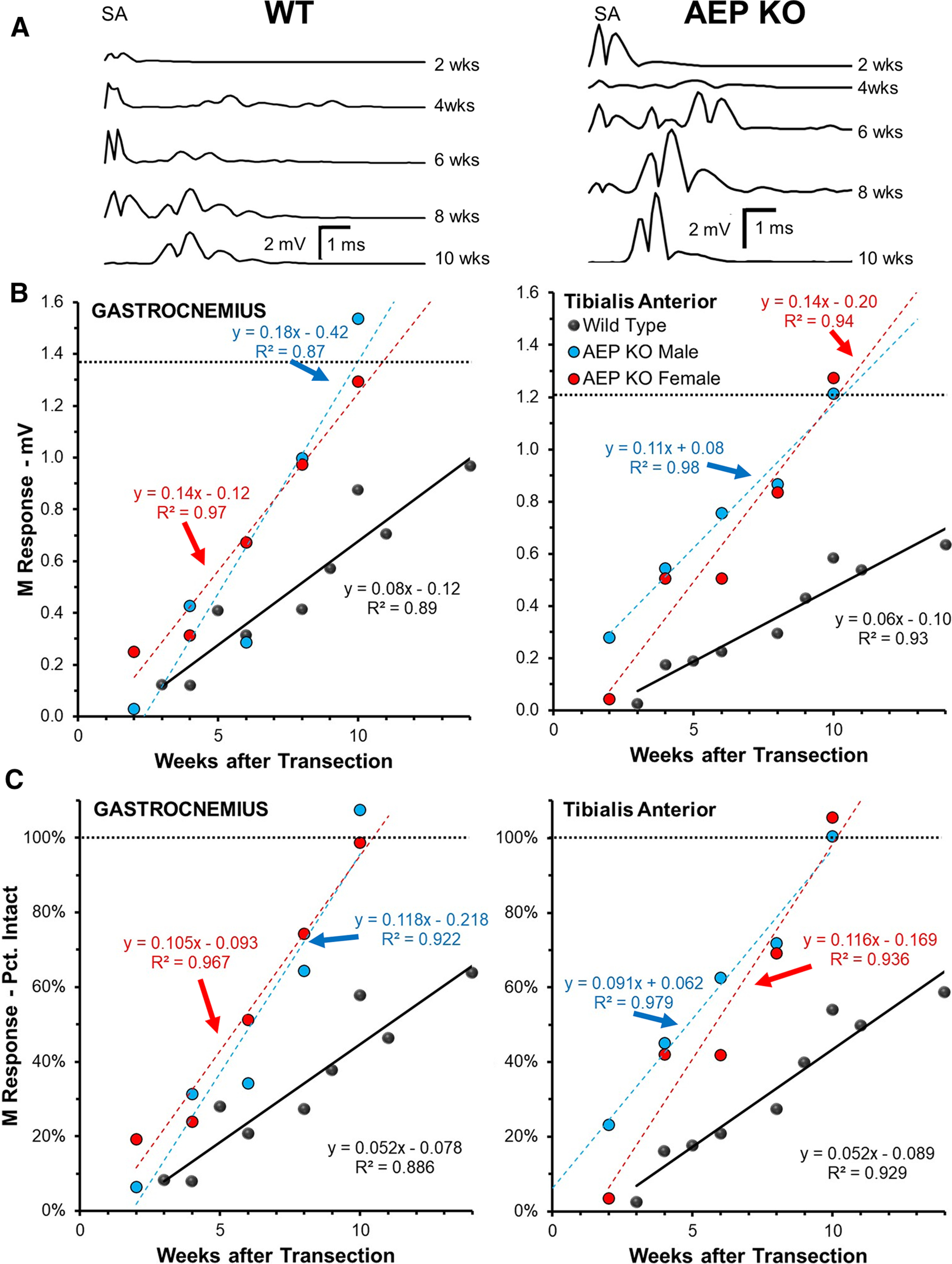
Recovery of evoked muscle responses is accelerated in AEP KO mice. ***A***, Examples of full wave rectified maximal direct muscle (M) responses to sciatic nerve stimulation recorded from GAST muscles at different times after transection and repair of the sciatic nerve. Traces on the left are from a single WT mouse. Those on the right are from a single AEP KO mouse. ***B***, Maximum amplitude M responses were measured in GAST (left) and TA (right) at different times after sciatic nerve transection and repair. Data were fit with linear least-square regression lines. Regression and correlation coefficients are shown for each fitted line. ***C***, Amplitudes of recorded responses in ***B*** were scaled to preinjury values in each mouse and re-plotted. Regression and correlation coefficients are shown for each fitted line.

To evaluate whether the enhancement of motor axon regeneration observed above resulted in enhanced functional recovery, compound muscle action potentials (direct muscle or M responses) were recorded from GAST and TA muscles in response to proximal sciatic nerve stimulation. Examples of maximum amplitude M responses (M_MAX_) recorded from GAST muscles in a WT and an AEP KO mouse at different times after sciatic nerve transection and repair are shown in Figure 5*A*. Changes in M_MAX_ amplitudes over postinjury time are shown for both GAST and TA muscles in groups of WT mice and male and female AEP KO mice in Figure 5*B*. M_MAX_ amplitudes recorded at different times after sciatic nerve transection and repair also were scaled to the amplitudes of responses recorded before injury to demonstrate the rate of recovery of functional muscle reinnervation. These data are shown in Figure 5*C*. In both muscles studied, a linear relationship between M response amplitude and postinjury time was found whether the data were studied as raw voltages (Fig. 5*B*) or scaled (Fig. 5*C*). Data in each group were fit using least squares linear regression methods and the slopes of the lines fitted to the data were compared between groups using multiple linear regression analysis. Data from male and female AEP^−/−^ mice were compared separately to that of a mixed sex population of WT controls.

Slopes of the lines fitted to the data were significantly different from zero for all three comparison groups and both (raw and scaled) methods of analysis (*p* < 0.01 for both muscles in all groups). Slopes of lines from AEP^−/−^ mice of both sexes were roughly twice those from WT mice. These differences were significant (*p* < 0.01 for both GAST and TA, both raw and scaled). No significant sex differences were found in the slopes of the lines fitted to the data for AEP^−/−^ mice, for GAST (*p* = 0.35 for raw, *p* = 0.59 for scaled) or TA (*p* = 0.24 for raw and scaled). The rate of recovery of the M response in both muscles in the AEP^−/−^ mice was thus approximately twice that observed in WT controls.

### Axon regeneration is not further enhanced by ES in AEP^−/−^ mice

In AEP^+/+^ and AEP^−/−^ mice, we measured M responses in intact mice and then cut sciatic nerves, unilaterally, and repaired them immediately after application of 1 h of continuous 20-Hz supramaximal ES. Four weeks later, we evaluated the extent of restoration of the M response elicited by stimulation of motor axons that had successfully regenerated and reinnervated the TA and GAST muscles. Comparisons of scaled M response amplitudes between males and females, including data from untreated WT and AEP^−/−^ mice at this four-week survival time (from data presented above) were made using a two factor (treatment vs sex) ANOVA. A significant effect of treatment (*F*_(7,32)_ = 6.48, *p* < 0.01) was found, but not of sex (*F*_(3,32)_ = 1.586, not significant) or interaction (*F*_(21,32)_ = 0.113, not significant), so that data from males and females were combined.

**Figure 6.**
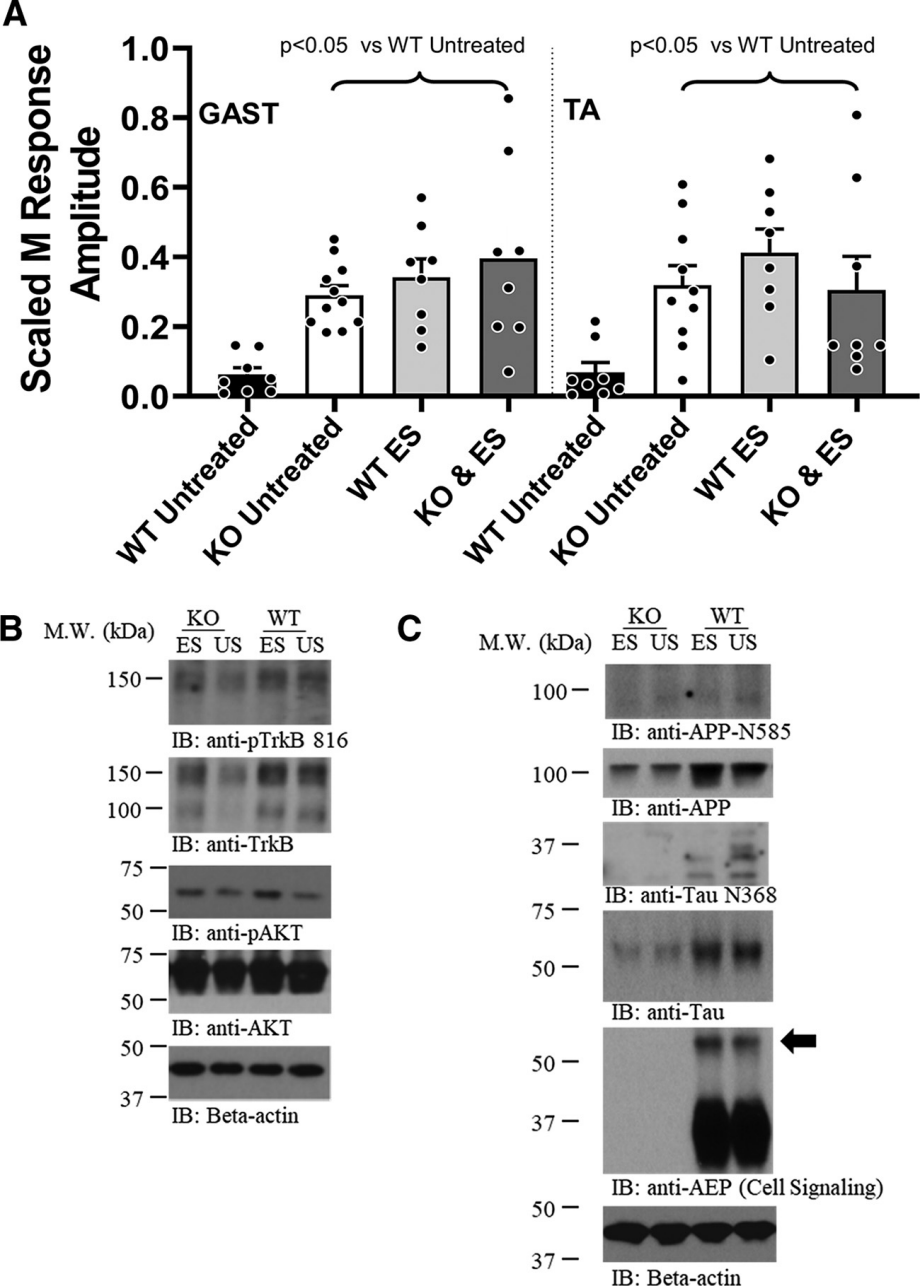
***A***, Effects of ES on recovery of direct muscle (M) response amplitude in AEP KO mice. Mean (±SEM) scaled M response amplitudes recorded from GAST (left) and TA (right) muscles four weeks after sciatic nerve transection and repair. Both AEP KO mice and WT mice were studied. Mice of each genotype either were treated at the time of nerve repair with 1 h of 20-Hz ES of the proximal nerve stump or left untreated. ***B***, Effects of ES on BDNF-TrkB expression in WT and AEP KO mice. Immunoblots from sciatic nerves 7 d following nerve transection and repair were probed for two markers of BDNF-TrkB signaling: phosphorylation of the TrkB receptor at Y816 and phosphorylation of the downstream effector, AKT. IR to both is increased in ES mice relative to untreated (UT) controls in both WT and AEP KO animals. ***C***, Effects of ES on AEP cleavage of APP and Tau. Immunoblots from animals used in panel ***C*** were probed with antibodies to AEP, full-length APP and Tau, and the AEP cleavage products of these two proteins, APP N585 and Tau N368. The solid arrow points to the greater amount of IR to inactive AEP in the ES mouse.

Results of this analysis are shown in Figure 6*A*. Significant effects were found for both GAST (*F*_(3,31)_ = 7.05, *p* < 0.01) and TA (*F*_(3,30)_ = 4.67, *p* < 0.01). Based on *post hoc* paired testing, treatment of WT mice with ES at the time of nerve repair resulted in significantly (*p* < 0.01) larger M responses in both GAST and TA than found in US WT mice. This degree of enhancement of M response recovery was comparable to that found in US AEP^−/−^ mice. In AEP^−/−^ mice treated with 1 h of ES, scaled M response amplitudes in GAST and TA also were significantly greater than US WT controls (*p* < 0.05), but not significantly different from US AEP^−/−^ mice or WT mice treated with ES at the time of nerve repair.

To investigate whether BDNF-TrkB signaling is up regulated similarly in cut and repaired nerves after ES from both genotypes, we harvested nerve repair sites in WT and AEP KO mice one week after sciatic nerve transection and repair and treatment with 1 h of 20-Hz ES or US controls, and processed lysates from the nerves for detection of two key downstream markers of this signaling using immunoblots. IR to both phosphorylated AKT and TrkB phosphorylated at Y816 was elevated following ES, relative to US mice, in both WT and AEP KO mice (Fig. 6*B*), indicating that the effectiveness of ES in driving this signaling pathway is similar in mice of both genotypes. Others have shown that ES produces effects on motoneurons and DRG neuron expression of BDNF and TrkB mRNA with a time course compatible with these observed effects (Al-Majed et al., 2004; Gordon et al., 2009).

We also evaluated the effects of ES on AEP activation and its downstream effects on Tau and APP. Results of this analysis are shown in Figure 6*C*. In immunoblots of nerve extracts from AEP KO mice, we found significant reduction in IR to full-length APP and full-length Tau in both ES and US animals. This reduced expression is likely because of a diminished activation of CCAAT/enhancer-binding protein β (C/EBPβ) in the absence of AEP. This transcription factor mediates expression of both APP and Tau (Zhang et al., 2020). In AEP KO mice, little or no IR to APP N585 or Tau N368, products of AEP destruction of APP and Tau, respectively, was observed in either ES or US mice. In WT mice, treatment with 1 h of ES one week earlier resulted in less cleavage and activation of AEP (i.e., more IR at ∼60 kDa; Fig. 6*C*, arrow) and reduced IR to APP N585 and Tau N368 (Fig. 6*C*), relative to US controls.

## Discussion

Poor functional recovery after PNI is often attributed to the slow and inefficient process of axon regeneration (Gordon et al., 2009). We hypothesized here that AEP activity could be a major contributor to this poor recovery. In cultured neurons and mouse brains, the axonal microtubule-associated protein, Tau, is cleaved by AEP, removing its microtubule-binding domain. Cleavage of Tau by AEP thus might be expected to impede the elongation of regenerating axons (Biswas and Kalil, 2018). APP is also cleaved by AEP and because of its association with neurite elongation and axon guidance (Southam et al., 2019), its degradation by AEP might be expected to slow or disrupt axon regeneration after PNI. Thus, we wanted to investigate whether AEP activity after PNI could inhibit axon regeneration, as predicted from our hypothesis. We found that AEP expression and enzymatic activity is strongly increased in the regions of injured nerves in which regenerating axons from the proximal stump of the cut nerve are navigating into a regeneration pathway in the distal nerve segment. The increased enzymatic activity of AEP is initiated nearly immediately after nerve injury and persists for at least four weeks. In injured nerves this increased AEP expression results in a significant cleavage of Tau at its microtubule-binding domain, thereby eliminating its stabilizing effect. The newly expressed AEP also cleaves APP in these nerves at a site that could lead to an inhibition of axon regeneration. Based on these findings, we predicted that reducing or eliminating AEP enzymatic activity would promote axon regeneration after PNI.

Using AEP^−/−^ mice, we showed that axon regeneration was markedly improved both in the absence of this enzymatic activity or when it is reduced in heterozygous animals. Profiles of regenerating axons were longer in AEP KO mice and neuromuscular responses, a functional measure of successful motor axon regeneration, were restored faster in the absence of AEP. When AEP was eliminated only from the pathway surrounding the regenerating axons, no significant effect on axon elongation was found, suggesting that the enhancement observed in AEP^−/−^ mice is targeted toward the regenerating axons or perhaps host immune cells migrating into the injury site, and not on repair Schwann cells. Neurites extending from cultured adult DRG cells from AEP^−/−^ mice after only 72 h *in vitro* were significantly longer than those extending from neurons from AEP^+/+^ mice. Although these cultures consist of some cells other than neurons, we speculate that it is unlikely that eliminating AEP from these non-neuronal cells is the primary source of this exuberant outgrowth. However, unlike the results of the *in vivo* experiments, neurite length *in vitro* was not increased in cultures from AEP^+/−^ mice, suggesting that, in the absence of contributions from non-neuronal cells, even some AEP expression may inhibit axon growth significantly.

Whether the enhancement of axon regeneration we observed in the AEP^−/−^ mice is solely attributable to a preservation of Tau to stabilize nascent microtubules or whether it also could be attributed to a reduced cleavage of APP remains to be investigated. AEP proteolytically cleaves APP at N373 and N585, in its ectodomain, and this affects the rate of subsequent BACE1 cleavage of the resultant substrate (Zhang et al., 2015). One result of BACE1 processing is the formation of Aβ, a key pathogen in the development of AD. It has been shown that Aβ induced growth cone collapse in cultured mouse cortical neurons (Kuboyama, 2018). Such a role for Aβ in axon regeneration in peripheral nerves is consistent with previous results on the role of BACE1 in axon regeneration in peripheral nerves. Blocking its activity enhances regeneration (Farah et al., 2011) and over expressing BACE1 inhibits regeneration (Farah, 2012; Tallon et al., 2017). These studies pointed to other important BACE1 substrates, like the cell adhesion molecules, L1 and its close homolog, CHL1, but we suggest that BACE1 cleavage of APP, made easier by prior AEP activity, ought to be considered, too. Sorting out these potential roles of AEP will be a goal of future experimentation.

Our recent finding (Xia et al., 2020) that TrkB is a substrate for AEP cleavage is also of interest in this context. Cleavage of TrkB at N365 and N486 residues by AEP suppresses BDNF neurotrophic effects. Activated TrkB binds to and phosphorylates APP on its intracellular domain at Y687 and mediates its subcellular distribution, repressing Aβ production. Cleavage of TrkB by AEP leads to APP endosomal/TGN translocation and an escalation of Aβ production (Xia et al., 2020). At least a part of the enhancement of axon regeneration observed in AEP KO mice could be attributed to an increase in this neurotrophin-based inhibition of production of the growth inhibiting Aβ.

In the brain, AEP is inhibited by phosphorylation at T322 by Akt, a downstream effector of neurotrophin signaling (Wang et al., 2018). Application of 1 h of 20-Hz ES to injured peripheral nerves is an activity-dependent treatment known to enhance axon regeneration in a manner that requires neuronal BDNF-TrkB signaling in the regenerating axons (Gordon and English, 2016). We show here that despite the predicted decrease in the breakdown of Tau and APP produced by treatment with ES in WT mice, and although TrkB phosphorylation at Y816 and phosphorylation of Akt, known markers of BDNF-TrkB signaling, are increased with ES in both genotypes, the extent of axon regeneration after PNI found in AEP^−/−^ mice was not increased by ES. In addition, when the restoration of M responses in AEP^−/−^ mice was compared with a published study of the effects of treatments with the small molecule TrkB agonist, 7,8-dihydroxyflavone (English et al., 2013), a nearly identical increased rate of restoration of neuromuscular function was found. Based on these observations, we hypothesize that the effectiveness of treatments that activate the TrkB receptor, including ES or treadmill exercise (English et al., 2011) or application of small molecule TrkB agonists (English et al., 2013) is, at least in part, via their inhibition of AEP.

The results presented above also lead us to ponder as to what role AEP might be playing during axon regeneration. It is clearly increased dramatically after PNI and just as certainly has an anti-growth effect on regenerating axons. It is possible that this expression of active AEP might be related to pathfinding by regenerating axons. For axon regeneration to be successful after injuries like the ones used in the present study, axons proximal to the injury need to find their way into a suitable regeneration pathway within endoneurial tubes in the distal nerve segment and once in that pathway, elongate toward a target. Achieving this connectivity may involve repeated elongation and retreating of neurites until an appropriate path is found, as well as to avoid debris related to anterograde axonal degeneration once in the pathway. Such movements by early regenerating axons have been proposed to explain fluctuations in rates of elongation of individual axons in mice (Kang and Lichtman, 2013). One role of the increased expression of AEP during this process might be to facilitate the retraction portion of this scenario. Whether or not this speculation survives future rigorous experimental testing, we believe that inhibiting AEP should be viewed as a promising therapeutic target for treating PNIs.

## Synthesis

Reviewing Editor: Jeffery Twiss, University of South Carolina Decisions are customarily a result of the Reviewing Editor and the peer reviewers coming together and discussing their recommendations until a consensus is reached. When revisions are invited, a fact-based synthesis statement explaining their decision and outlining what is needed to prepare a revision will be listed below. The following reviewer(s) agreed to reveal their identity: Dianna Willis. Note: If this manuscript was transferred from JNeurosci and a decision was made to accept the manuscript without peer review, a brief statement to this effect will instead be what is listed below.

Regeneration following peripheral never injury is limited. This manuscript identifies a novel mechanism (asparagine endopeptidase [AEP]) that might, in part, underlie this limited regeneration, and may point to a potential therapeutic target to enhance functional regeneration. The findings may support AEP inhibition as a therapeutic target for improving axonal regeneration after injury. The authors demonstrate a significant and sustained activation of AEP activity following nerve injury, supported by increase AEP levels and increased levels of AEP-leaved fragments of Tau and APP. They further show that AEP null mice exhibit increased axonal regeneration in vivo following injury compared to wild type mice, and enhanced functional muscle reinnervation, as well as increased neurite outgrowth when cultured in vitro. Finally, they show that using electrical stimulation paradigms that have previously been shown to enhance regeneration in wild type animals, AEP null mice do exhibit further enhancement of regeneration, suggestion that electrical stimulation is acting in an AEP-dependent manner. Overall, the reviewers and reviewing editors feel that is a well-written manuscript describing a potential target for altering peripheral nerve regeneration following injury that would be of interest to eNeuro readership. The experiments conducted in this study provide promising preliminary evidence, but in some cases lack clear controls. While we think it will likely be of significant interest to readers, there are a few questions that, if adequately addressed, would substantially improve the manuscript.

Major Issues:

1. The authors propose that electrical stimulation (ES) may be acting by inhibiting AEP activity to improve regeneration. They point to prior data that ES requires neuronal BDNF-TrkB signaling and that in the brain AEP is inhibited by phosphorylation of Akt. I think that the link between ES, AEP activity, and regeneration needs to be further demonstrated here - in other words, the case that AEP is a good therapeutic target for enhancing axonal regeneration is not convincingly demonstrated. It should be relatively easy to take lysates from wild type animals that have been subjected to the regeneration-enhancing ES treatment and look for differences in AEP levels and Tau and APP cleavage levels compared to injured untreated animals. This would more directly like the effects of ES on regeneration to altered AEP activity, further strengthening this as a relevant target for regeneration. Also the possibility for other targets for AEP is not discussed - the authors should of some speculation on the need to link AEP to other regeneration relevant substrates.

The authors could also consider western blotting to interrogate whether BDNF-TrkB signaling is up regulated after ES of nerves from both genotypes. In addition, some loss/gain of function of this BDNF-TrkB signaling pathway in AEP KO neurons would support the argument more, but is likely beyond what is needed to sufficiently substantiate the ES data.

2. Do the data in Figure 3 point to a difference in the number of regenerating axons? It appears as if, while the AEP null animals have enhanced regeneration in terms of length of regenerating axons, there may be more axons that sprout in the AEP wild type animals. In other words, the axons may be shorter but there are more of them that are regenerating overall. Do the data bear this out? Also, showing reinnervated NMJ staining with bungarotoxin would strengthen the regeneration/electrophysiology data.

Minor Comments:

1. Is the blot shown in Figure 1C the best representative of the overall data? The 14 d band is relatively low, and this is evidently not indicative of the overall response as indicated in the quantitation in Figure 1D. Additionally, β-actin loading controls are not included in the Figure, and it is what loading controls were used in C-F.

2. The authors indicate that there is no N585 cleavage fragment of APP in intact nerves, yet the male sample in Figure 1E shows a prominent band.

3. Have the authors considered the opposite mixed host:graft to those shown in Figure 3? Is a AEP null host sufficient to drive enhanced regeneration even in a AEP containing wild type graft?

4. For CMAP data, raw amplitude values should be included, and representative traces should be shown.
